# The past, present and future of neutralizing antibodies for hepatitis C virus

**DOI:** 10.1016/j.antiviral.2014.02.013

**Published:** 2014-05

**Authors:** Jonathan K. Ball, Alexander W. Tarr, Jane A. McKeating

**Affiliations:** a School of Life Sciences and The Nottingham Digestive Diseases Centre Biomedical Research Unit, University of Nottingham, Queens Medical Centre, Nottingham NG7 2UH, United Kingdom; b Viral Hepatitis Research Group and Centre for Human Virology, Institute for Biomedical Research, University of Birmingham, Birmingham B15 2TT, United Kingdom

**Keywords:** Hepatitis C, Neutralization, Epitope, Transmission, HCV E2 core

## Abstract

Hepatitis C virus (HCV) is a major cause of liver disease and hepatocellular carcinoma worldwide. HCV establishes a chronic infection in the majority of cases. However, some individuals clear the virus, demonstrating a protective role for the host immune response. Although new all-oral drug combinations may soon replace traditional ribavirin–interferon therapy, the emerging drug cocktails will be expensive and associated with side-effects and resistance, making a global vaccine an urgent priority. T cells are widely accepted to play an essential role in clearing acute HCV infection, whereas the role antibodies play in resolution and disease pathogenesis is less well understood. Recent studies have provided an insight into viral neutralizing determinants and the protective role of antibodies during infection. This review provides a historical perspective of the role neutralizing antibodies play in HCV infection and discusses the therapeutic benefits of antibody-based therapies. This article forms part of a symposium in *Antiviral Research* on “Hepatitis C: next steps toward global eradication.”

## Highlights

• Recent studies have provided insight into the protective role of neutralizing antibodies in hepatitis C.
• Neutralizing antibodies show broad reactivity for diverse HCV genotypes.
• Recombinant HCV glycoproteins can elicit neutralizing antibodies.
• The HCV E2 core structure can inform rational design of immunogens.

## 1 Introduction

Hepatitis C virus (HCV) has established chronic infection in approximately 170 million people worldwide and can lead to cirrhosis and hepatocellular carcinoma (HCC). Antiviral therapies have largely relied on interferon-based regimes that were poorly tolerated and ineffective in the majority of patients (Jesudian et al., 2013). It is unclear whether the new battery of direct acting antiviral therapies will cure hepatitis C in all cases, especially those infected with genotype 3 viruses or with co-morbidities, such as cirrhosis and HIV co-infection (Lange et al., 2014), highlighting the need for alternative immune-based therapies. At present there is no prophylactic or therapeutic HCV vaccine. HCV is amongst the few human pathogenic viruses that can establish a chronic infection or be cleared, demonstrating a protective role for the adaptive immune response in some individuals. Our current goal is to understand the determinants of a protective immune response and whether recombinant vaccines can induce such responses.

## 2 *In vitro* systems to measure HCV-specific neutralizing antibodies

Prior to the development of *in vitro* infection systems, the neutralizing potential of HCV-specific antibodies were evaluated using “neutralization of binding” assays (NOB), where antibodies were screened for their ability to prevent recombinant viral E2 glycoprotein binding to mammalian cells (Rosa et al., 1996). Baumert and colleagues developed a recombinant baculovirus system to express the HCV structural proteins which formed viral-like particles (VLPs) (Baumert et al., 1998) to study antibody reactivity and inhibition of VLP-cell interactions (Baumert et al., 2000). However, the discovery that lentiviral pseudoparticles expressing HCV glycoproteins (HCVpp) were infectious for hepatocytes and hepatoma cell lines (Bartosch et al., 2003b; Hsu et al., 2003) superseded these model systems and enabled studies to unravel the mechanism of HCV entry and to measure functional neutralizing antibody responses for the first time.

HCV encodes two envelope glycoproteins, E1 and E2, both of which are required for pseudoparticle infectivity. HCVpp infect primary human hepatocytes and hepatoma cell lines via a clathrin mediated endocytosis (Blanchard et al., 2006; Meertens et al., 2006) that is dependent on four essential host cell molecules: tetraspanin CD81; scavenger receptor class B member I (SR-BI) and tight junction proteins claudin-1 and occludin (Meredith et al., 2012; Zeisel et al., 2013). The HCVpp system has enabled the screening and identification of polyclonal sera (Bartosch et al., 2003a,c; Flint et al., 2004; Logvinoff et al., 2004; Sung et al., 2003; Yu et al., 2004) and monoclonal antibodies (Giang et al., 2012) that inhibit infection, demonstrating the cross-reactive nature of neutralizing antibody responses that are independent of the infecting or immunizing viral genotype, providing an impetus for developing antibody based therapeutics.

Early studies with the HCVpp system suggested that neutralizing antibodies were frequently observed in chronically infected subjects, raising the question as to how the virus can persist in the face of this response. However, serum antibodies are generally screened for the ability to neutralize a limited number of viral genotypes (Bartosch et al., 2003a; Broering et al., 2009). Recent studies using HCVpp expressing a panel of glycoproteins cloned from clinical material demonstrate differences in sensitivity to antibody neutralization, in contrasts the most commonly used H77c viral strain was easily neutralized by the majority of sera (Tarr et al., 2011; Osburn et al., 2014).

The discovery that the JFH-1 strain of HCV could generate infectious particles in cell culture (HCVcc) revolutionized the viral hepatitis field and allowed investigators to study the sensitivity of authentic viral particles to antibody-dependent neutralization (Lindenbach et al., 2005; Wakita et al., 2005; Zhong et al., 2005). To date, HCVcc has been reported to be neutralized *in vitro* by E2-specific antibodies derived from human sera (Lindenbach et al., 2005; Zhong et al., 2005), polyclonal Ig preparations derived from E1E2 immunized mice and guinea pigs (Stamataki et al., 2007) and by a diverse array of glycoprotein-specific monoclonal antibodies (mAbs) (Johansson et al., 2007; Keck et al., 2008; Law et al., 2008; Meunier et al., 2008; Pedersen et al., 2013; Perotti et al., 2008). The JFH-1 system can be modified to study the properties of genetically diverse viruses by the generation of chimeric clones encoding the structural proteins (core, E1, E2 and p7) and part of the non-structural protein 2 (NS2) of all major genotypes. Chimeras constructed using genotype 2 structural proteins replicate with similar kinetics to wild type virus without cell culture adaptation and have recently been used to confirm that cell entry mediated by patient-derived E1E2 is relatively resistant to neutralization by polyclonal serum (Pedersen et al., 2013).

The JFH-1 system has also been used as a backbone to construct inter-genotype chimeras, but these often show poor replication kinetics and acquire cell-culture adaptive mutations (Gottwein et al., 2009; Pietschmann et al., 2006). There is emerging evidence that at least some culture-adaptive mutations render the strains more sensitive to antibody neutralization (Dhillon et al., 2010; Grove et al., 2008). Therefore, a HCV-based single-cycle infection system, particularly one that could be complemented with E1E2 cloned directly *ex vivo*, would provide a more robust method to study antibody neutralization. Trans-complementation of HCV replicons with plasmids encoding the HCV structural proteins results in the production of infectious particles containing a packaged replicon genome (Adair et al., 2009). However, the relatively low virus titers produced limits the general applicability of this system.

A small animal model capable of supporting the complete replicative cycle would be invaluable to study the role of antibodies in HCV infection (Billerbeck et al., 2013; Mailly et al., 2013). The uPA-SCID mouse model uses immunosuppressed mice transplanted with human hepatocytes, which renders them susceptible to HCV infection (Mercer et al., 2001). Generation of chimeric livers is technically difficult and the mice are immunodeficient, limiting studies on host adaptive immunity. However, this model has been invaluable to confirm the efficacy of passively transferred antibodies to protect animals against challenge virus (Law et al., 2008; Vanwolleghem et al., 2008). More recently, transgenic immunocompetent mice expressing the essential HCV entry factors were reported to support HCV replication and administration of adenoviral expressed HCV glycoproteins induced antibody responses that limit infection (Dorner et al., 2013, 2011).

## 3 Do neutralizing antibodies influence HCV replication?

Due to the asymptomatic nature of acute HCV infection, identifying and studying patients in the early phase of infection is difficult. HCV infected individuals frequently have detectable RNA levels as early as one week following infection, however adaptive immune responses against the virus are not detected for several months (Chen et al., 1999). Several studies of acute HCV infection demonstrate that a broad and potent T cell response is important for virus clearance (reviewed in (Neumann-Haefelin and Thimme, 2013; Rehermann, 2013) and that the rapid induction of cross-reactive nAb responses associates with spontaneous recovery (Dowd et al., 2009; Pestka et al., 2007; Osburn et al., 2014). Limited studies of HCV evolution during acute infection show that resolving patients have stable HVR1 sequences, whereas chronically infected subjects show more noticeable HVR1 sequence change (Farci et al., 2000; Ray et al., 1999). The role of the humoral response in selecting viral diversity, particularly in the HVR, was reinforced by reports that HCV-infected subjects with hypogammaglobulinemia showed reduced rates of nucleotide substitution in the HVR compared to controls (Booth et al., 1998). The authors propose that the HVR serves as a “viral decoy”, directing the immune system away from viral epitopes potentially less capable of rapid change and towards those where faster rates of evolution can be tolerated (Liu et al., 2010; Mondelli et al., 2001; Ray et al., 1999; von Hahn et al., 2007).

The earliest studies on HCV specific neutralizing antibody responses were carried out with chimpanzees, showing that sera from infected animals could neutralize virus infectivity *in vitro* and subsequently protect chimpanzees against HCV challenge (Farci et al., 1996). In a separate study, chimpanzees immunised with E1E2 envelope glycoproteins elicited an antibody response that partially protected against experimental challenge with autologous HCV (Choo et al., 1994). More recently, transfusion of human monoclonal antibody (HCV1) mapping to E2 amino acids 412–423 protected a naïve chimpanzee from HCV challenge and reduced viral RNA levels in an acutely infected animal (Morin et al., 2012). Evidence that antibodies can protect humans arose from a retrospective cohort study of patients receiving polyclonal immunoglobulin against hepatitis B virus surface antigen (HBIG). Patients who received HBIG prior to the introduction of routine screening of blood donors for HCV infection were less likely to acquire HCV than those who received HBIG screened for HCV (Yu et al., 2004). Anti-HCV antibodies were detected in HCV-negative patients who had undergone HBIG treatment, suggesting a passive transfer of anti-HCV antibodies in HBIG to the recipient (Feray et al., 1998). This highlights the potential use of therapeutic antibodies for the prevention of HCV infection, especially in the liver transplant setting where early clinical trials were disappointing (Schiano et al., 2006; Davis et al., 2005) however a recent study reported that a humanized monoclonal antibody MBL-HCV1 delayed HCV RNA kinetics (Chung et al., 2013).

Even in chronically infected individuals, there is evidence that antibodies may partially control HCV replication. Firstly, hypogammaglobulinaemic individuals exhibit a marked rapidity and severity in disease progression (Bjoro et al., 1994). Secondly, B cell depletion during rituximab therapy leads to an increase in peripheral viral load, which returns to normal after cessation of therapy (Ennishi et al., 2008). Zibert et al. reported that 43% of individuals who spontaneously resolved infection had antibodies specific to the E2 hypervariable (HVR) region within the first 6 months of infection, compared to only 13% of patients who failed to clear infection (Zibert et al., 1997). In contrast, there were no significant differences between patient groups with respect to the time of emergence of antibodies to HCV core or non-structural proteins. Other investigators reported an early emergence of HVR1-specific antibodies in a cohort of subjects infected during haemodialysis who resolved infection (Allander et al., 1997). Since the HVR1 is proposed to be a target for neutralizing antibodies (Farci et al., 1996; Kato et al., 1993), several studies suggest a role of anti-HVR1 antibodies in selecting viral variants to escape the humoral response (Booth et al., 1998; Liu et al., 2010; Ni et al., 1999; van Doorn et al., 1995; von Hahn et al., 2007). A recent case study demonstrated that spontaneous clearance of chronic HCV infection associated with the appearance of neutralizing antibodies and a reversal of T-cell exhaustion (Raghuraman et al., 2012). It is likely that during chronic infection there is a dynamic interplay between host and virus such that mutations that lead to immune escape may also reduce viral fitness.

## 4 Fc-effector function of antibodies

The antibody Fc region plays an essential role in controlling chronic viral infections (Hessell et al., 2007) and can mediate antibody dependent cellular cytotoxicity (ADCC), complement-dependent cytotoxicity (CDC) or antibody-dependent phagocytosis by cells possessing Fcγ receptors. Our level of understanding on the role of antibody Fc-mediated antibody activity in controlling HCV replication is limited. A single report suggests that antibodies targeting HCV E2 can mediate ADCC (Nattermann et al., 2005). A significant role for complement to limit HCV infection is emerging. Recent studies suggest that HCV has evolved to evade complement mediated lysis, via down-regulation of complement factors (Banerjee et al., 2011; Kim et al., 2013; Mazumdar et al., 2012) and incorporation of host CD55 and CD59 into virus particles (Amet et al., 2012; Ejaz et al., 2012; Mazumdar et al., 2013). Antibody-mediated neutralization of HCV *in vitro* is enhanced in the presence of complement (Machida et al., 2008; Meyer et al., 2002), suggesting a potential role for antibody isotypes to play a role in viral clearance *in vivo*. Antibody-dependent phagocytosis of HCV by anti-E2 antibodies was reported to occur and was enhanced by complement (Eren et al., 2006).

In addition to contributing to clearance, Fc-mediated effects may be detrimental and non-neutralizing monoclonal antibodies have been reported to enhance HCV pseudotype infectivity by promoting interaction with FcγRII and FcγRI on target cells (Meyer et al., 2008). Furthermore, polyclonal serum antibodies isolated from the sera of chronic HCV infected patients have been reported to enhance infection (Tarr et al., 2011). The interplay between protection and enhancement of infection has implications for successful antibody-based therapy and highlights the need to study the impact of isotype and Fc-dependent functions on the antiviral activity of antibodies targeting the HCV glycoproteins.

## 5 Neutralizing antibody epitopes

**Fig. 1:**
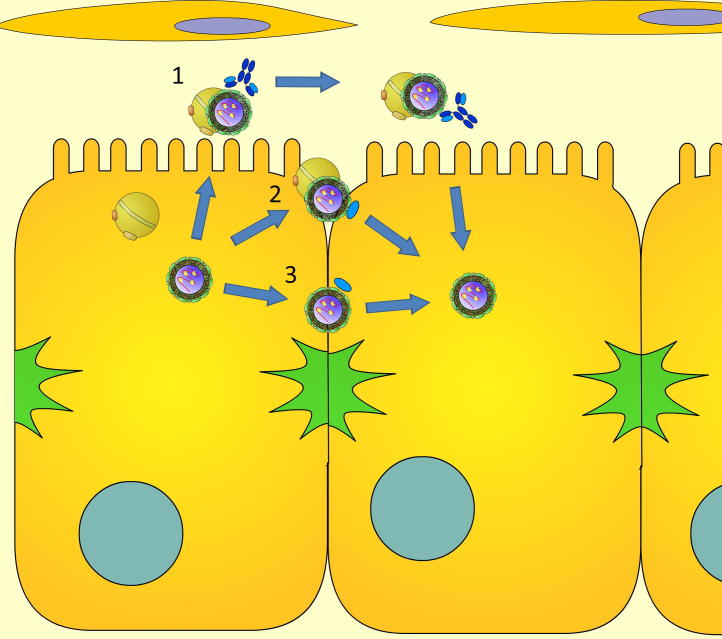
Neutralizing activity of anti-HCV antibodies and nanobodies. HCV particles circulate as lipoviral particles (LVPs) in the blood, in complex with low-density lipoproteins. Antibody epitopes are accessible on these LVPs. In an established infection, naïve cells can be infected by extracellular virus or by direct cell–cell transmission between adjacent cells. Antibody-mediated neutralization can occur as particles are released from infected cells (1), preventing HCV entry into naïve hepatocytes. While antibody based therapy appears to inhibit the extracellular route of infection, nanobodies (blue oval) have recently been demonstrated to inhibit the direct transmission of HCV between cells. It remains to be determined if this neutralizing activity is mediated via the blockade of cell-tethered virus (2), or direct inhibition of virus transmission between cell–cell junctions (3).

**Fig. 2:**
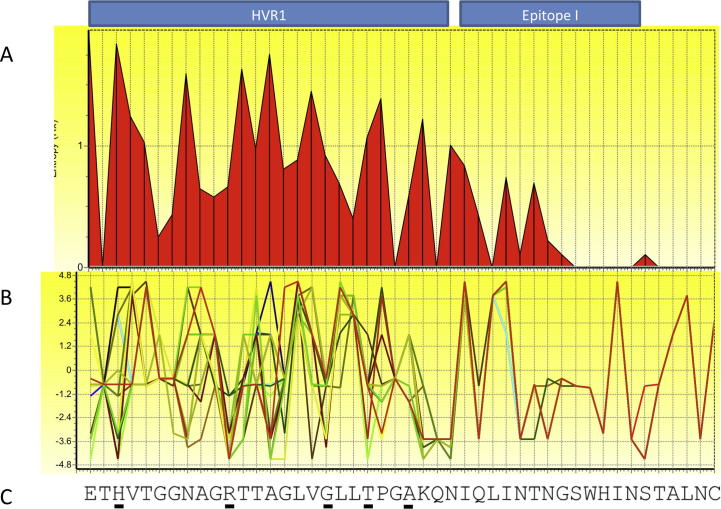
Variation in the HCV E2 glycoprotein N-terminus. Diversity of HCV E2N-terminal aa 384–429. (A) An entropy plot was performed to measure variability across viral strains – within HVR1 (aa 384–410), functionally conserved residues are observed at positions 385, 406 and 409. The well-described ‘Epitope I’ conserved region (Zhang et al., 2007) contains epitopes of neutralizing mAbs AP33, 3/11 (Tarr et al.) and HCV1 (Kong et al., 2012a,b). (B) A Kyte-Doolittle hydrophobicity plot reveals the hydrophobic nature of the HVR1 between patient sequences. (C) Residues responsible for cross reactivity of antibodies directed to the HVR1 are highlighted on HCV reference H77 strain. These residues are co-incident with regions possessing hydrophilic amino acids that are likely to be exposed on the surface of the virus particle.

nAbs exert their effect(s) by binding directly to virus particles and blocking subsequent interaction(s) with receptors or by inhibiting post entry events such as viral uncoating and subsequent replication (Burton, 2002). The former may occur by inducing conformational changes in the viral envelope that disable infection or by steric hindrance, physically shielding important viral interaction sites. The epitopes recognized by neutralizing antibodies map to the viral encoded E1E2 envelope glycoproteins and the majority of reported antibodies block CD81 receptor interaction (Fig. 1) (Hsu et al., 2003; Johansson et al., 2007; Keck et al., 2008, 2012; Meunier et al., 2008; Perotti et al., 2008). Early chimpanzee studies identified the HVR in the E2N-terminus as a major target for neutralizing antibodies. This region possesses multiple linear epitopes between amino acids (aa) 384–410 that are important for antibody recognition and binding scavenger receptor class B type I (SR-BI), a lipoprotein receptor molecule essential for HCV entry (Bartosch et al., 2003c; Scarselli et al., 2002). Antibodies targeting the HVR1 were observed *in vivo* (Kato et al., 1994, 1993; Weiner et al., 1992; Zucchelli et al., 2001); however not surprisingly anti-HVR antibodies show strain-specific neutralizing activity (Fig. 2). In an attempt to overcome their limited reactivity, Zucchelli and colleagues generated peptide mimics representing diverse HCV sequences that induced antibody responses capable of recognizing diverse patient derived variants (Zucchelli et al., 2001). Recent studies show that viruses lacking a HVR1 are more susceptible to neutralization by a panel of human mAbs and patient sera, suggesting that the HVR1 masks the E2-CD81 binding site (Bankwitz et al., 2010; Prentoe et al., 2011).

The lack of broadly neutralizing antibodies targeting the HVR1 led to the search for conserved epitopes. Whilst neutralizing antibodies targeting conserved epitopes overlapping the SR-BI binding site (Sabo et al., 2011), discontinuous residues not involved in CD81 binding (Giang et al., 2012) and E1 determinants (Meunier et al., 2008) have been described, the majority of neutralizing antibodies target the CD81 binding site (Johansson et al., 2007; Law et al., 2008; Owsianka et al., 2005, 2008; Perotti et al., 2008). Antibody competition studies provided the first insight into the region of E2 involved in CD81 binding and subsequent mutagenesis studies identified an essential role for residues W^420^, Y^527^, W^529^, G^530^ and D^535^ (Owsianka et al., 2006) and ^436^GWLAGLFY^443^ in binding CD81 (Drummer et al., 2006). Neutralizing antibodies targeting the CD81 binding site can be divided into three groups depending on whether they recognise: (i) linear epitopes located between E2 amino acids 412–423 (e.g. murine mAbs AP33, 3/11); (ii) conformational epitopes where key contact residues are located between residue 529 and 535 (e.g. human mAbs 1:7, A8, CBH2); or (iii) epitopes spanning these two CD81 binding regions (e.g. AR3A, AR3C, e137). Importantly, the most potent and broadly neutralizing murine antibodies target linear epitopes covering amino acid residues 412–423, whereas human antibodies to this region are rare (Tarr et al., 2007). In contrast, the majority of neutralizing human antibodies recognize conformation-sensitive epitopes centred on the key CD81 binding residues W^529^, G^530^ and D^535^ (Owsianka et al., 2006).

Antibodies targeting epitopes within the envelope glycoprotein E1 have been identified in some patients, but are generally rare (Pestka et al., 2007). This may reflect the technical difficulties in detecting anti-E1 responses, as the protein misfolds unless co-expressed with E2 (Dubuisson et al., 1994). Previous trials of E1 glycoprotein vaccine candidates induced antibody responses (Garrone et al., 2011; Leroux-Roels et al., 2004; Nevens et al., 2003) that had minimal effect on peripheral HCV RNA levels in chronically infected patients (Nevens et al., 2003). However, a recent study in chimpanzees showed that immunization with recombinant E1 protected animals against experimental infection with heterologous HCV (Verstrepen et al., 2011). Due to the limited understanding of the role and structure of E1 and how it interacts with E2, the mechanism of protection and whether it is antibody-dependent is unknown. It is important to note that E1 has been reported to contain a putative fusion peptide (Lavillette et al., 2007; Flint et al., 1999b) that may provide a target for neutralizing antibodies based on the HIV-1 and influenza literature (Karlsson Hedestam et al., 2008; Shui et al., 2009).

Zhang and colleagues reported the presence of anti-E2 antibodies binding aa 434–446 in patient and chimpanzee immune-sera that interfered with the activity of nAbs targeting epitopes located between residues aa 412–423 (Zhang et al., 2007, 2009). The authors suggest that the presence of such inhibitory antibodies may explain the failure of anti-HCV polyclonal immunoglobulin preparations to prevent HCV infection in the liver transplant setting (Davis et al., 2005). Subsequent studies by the same group, using murine monoclonal antibodies, argued that E2 aa 427–446 was targeted by interfering and non-interfering non-neutralizing antibodies. However, interference was only demonstrated with chimpanzee serum antibodies immunopurified with peptide aa-412–423 and furthermore the affinity the non-neutralizing antibodies for aa 427–446 peptide was low compared to the corresponding neutralizing antibodies targeting the same peptide (Duan et al., 2012). The interfering antibody hypothesis contradicts earlier observations made by Feray et al. where polyclonal immunoglobulins were found to protect against HCV infection (Feray et al., 1998). In addition, we (Tarr et al., 2012) and others (Keck et al., 2013) reported that human and murine antibodies targeting epitopes within aa 434–446 neutralize HCVpp and HCVcc and show additive neutralization with antibodies targeting aa 412–423.

## 6 Strategies for HCV to escape neutralizing antibodies

The most widely reported viral evasion mechanism is mutational escape. HCV contains a single-stranded positive-sense RNA genome that is replicated by a virus-encoded RNA-dependent RNA polymerase. This polymerase lacks proof-reading capabilities which, when coupled with the high replication rate of the virus, results in the generation of a diverse population of viral variants or quasispecies (Simmonds, 2004). This virus population can harbour neutralization escape variants that have a selective advantage over sensitive variants. Positively selected amino acids are located within and around known receptor- and neutralizing antibody-binding regions (Brown et al., 2005, 2007). E1E2 evolution is driven by the neutralizing antibody response and escape variants can become the dominant circulating strain (Dowd et al., 2009; Farci et al., 2000; von Hahn et al., 2007). Both von Hahn et al. and Dowd et al. reported that sequential sera show a limited ability to neutralize concurrent circulating viral strains but efficiently neutralize virus strains from earlier time points (Dowd et al., 2009; von Hahn et al., 2007). Concluding that a single viral envelope sequence is unlikely to represent the population of viruses within the liver.

The most broadly neutralizing antibodies reported to date target the CD81 binding site, and HCV has evolved various methods of shielding this region of the glycoprotein. E2 contains up to 11 potential N-linked glycosylation sites, nine of which are conserved across genotypes (>97%) (Helle et al., 2007). Glycans are important for the structure and function of glycoproteins and are critical for HCVpp entry into target cells (Goffard et al., 2005; Falkowska et al., 2007). Specific glycans are known to mask the CD81 binding site and mutation of these sites leads to increased CD81 binding and sensitivity to neutralization by patient sera and mAb (Falkowska et al., 2007; Helle et al., 2007, 2010; Pantua et al., 2013). Changes occur in the frequency and position of glycans on both HIV-1 gp120 and influenza HA glycoproteins, and these “evolving glycan shields” limit virus sensitivity to antibody neutralization (Abe et al., 2004; Wei et al., 2003). Whilst there is some variability in the location and number of glycosylation sites across different HCV E1E2 sequences (Helle et al., 2007), particularly in genotype 3 viruses (Brown et al., 2007) (Anjum et al., 2013; Humphreys et al., 2009), there is limited evidence for glycans to undergo significant intra-host evolution (Brown et al., 2007; Helle et al., 2007).

More recently, HCV was reported to transmit via cell-to-cell junctions, providing an additional mechanism to escape neutralizing antibodies (Brimacombe et al., 2011; Timpe et al., 2007; Witteveldt et al., 2009). Many enveloped viruses, including herpes simplex virus, human T cell lymphotropic virus, HIV and measles virus transmit via cell-to-cell junctions to evade host immune responses (Mothes et al., 2010). The exact mechanism of HCV cell-to-cell transmission is still unknown but is dependent on the same viral receptors and ApoE as extracellular virus particles. Tarr and colleagues recently reported that a nanobody targeting the E2-CD81 binding site could inhibit cell-to-cell infection, suggesting that transmitting virus is not located in a synapase that is inaccessible to antibodies and that the lower molecular weight of the nanobody may promote access to cell-tethered virus (Tarr et al., 2013).

## 7 Inducing protective immunity *in vivo*

The propensity for HCV to establish chronic infection, to re-infect previously exposed individuals (Aitken et al., 2008), to transmit directly by cell–cell routes *in vitro* (Brimacombe et al., 2011; Timpe et al., 2007) and to evolve neutralization escape variants (Shimizu et al., 1994) makes the development of a HCV vaccine a major challenge. However the existence of natural immunity to infection in some humans (Mehta et al., 2002) and chimpanzees (Bassett et al., 2001; Farci et al., 1992; Lanford et al., 2004; Weiner et al., 2001) is encouraging and suggests that the immune system can eliminate infection. Immunization of chimpanzees with recombinant preparations of HCV glycoprotein protected animals from challenge with autologous virus (Choo et al., 1994). Of note, in animals with reduced antibody responses (Choo et al., 1994) and those challenged with heterologous virus (Nattermann et al., 2005), sterilizing immunity was not achieved (Puig et al., 2004; Youn et al., 2005) however, the animals failed to progress to a chronic state of infection. Reports on the immunogenicity of recombinant HCV glycoproteins in mice and non-human primates are variable (Elmowalid et al., 2007; Jeong et al., 2004; Lechmann et al., 2001; Murata et al., 2003; Qiao et al., 2003; Stamataki et al., 2007), with higher titer antibody responses generally observed in mice.

Two recent publications demonstrate the value of using inactivated HCVcc (Akazawa et al., 2013) or virus-like particle pseudotyped with HCV glycoproteins (Garrone et al., 2011) to protect mice or macaques, respectively against HCV challenge. Importantly, vaccination of healthy volunteers with HCV-1 E1E2 glycoproteins elicited serum antibody responses that were able to neutralize heterologous HCVpp and HCVcc *in vitro* and were detectable one year post vaccination (Law et al., 2013; Stamataki et al., 2011), highlighting a number of promising vaccine candidate leads. Ideally, any future vaccine should induce both T cell and B cell immunity and studies to optimize a combined regimen including the recently reported adenovirus-based HCV T cell vaccine (Barnes et al., 2012) ∗with recombinant E1E2 protein boost will be valuable. Recent studies showing the efficacy of passive administration of polyclonal anti-HCV Ig (Meuleman et al., 2011) and anti-E2 neutralizing monoclonal antibodies (Law et al., 2008) in preventing HCV infection of mouse models, highlight the value of neutralizing monoclonal antibodies for therapeutic or prophylactic purposes.

## 8 HCV glycoprotein structure

Receptor and antibody mapping studies have increased our understanding of the topology of key antigenic and functional determinants of the HCV glycoproteins. Competition assays with a panel of neutralizing and non-neutralizing antibodies led to the development of a three-domain structure for E2 glycoprotein with domains A, B and C containing non-neutralizing and broad and restricted neutralizing epitopes, respectively (Keck et al., 2005, 2004). Additional studies highlighted the discontinuous nature of E2 CD81 binding site (Clayton et al., 2002; Flint et al., 1999a; Owsianka et al., 2001), that informed the development of alanine replacement glycoprotein panels that facilitated fine detail mapping of this key receptor binding site (Drummer et al., 2006; Owsianka et al., 2006; Rothwangl et al., 2008).

Whilst most potent and broad neutralizing antibodies recognize conformational epitopes a limited number have been shown to bind linear epitopes. One of the first conserved linear neutralizing determinants to be described was the region of E2 encompassing aa 412–423, that is targeted by murine AP33 (Owsianka et al., 2005) and 3/11 (Flint et al., 1999b) and human monoclonal antibodies HCV1 (Broering et al., 2009) and HC33 (Keck et al., 2013)). Our early studies demonstrated that antibodies targeting this region were capable of potent and broad neutralization (Owsianka et al., 2005; Tarr et al., 2006), so understandably this epitope has been the subject of interest in terms of vaccine design. Epitope mapping studies highlighted that each of these antibodies target overlapping, but distinct epitopes (Broering et al., 2009; Keck et al., 2013; Tarr et al., 2006) and this may explain their differing neutralizing potency and breadth.

**Fig. 3:**
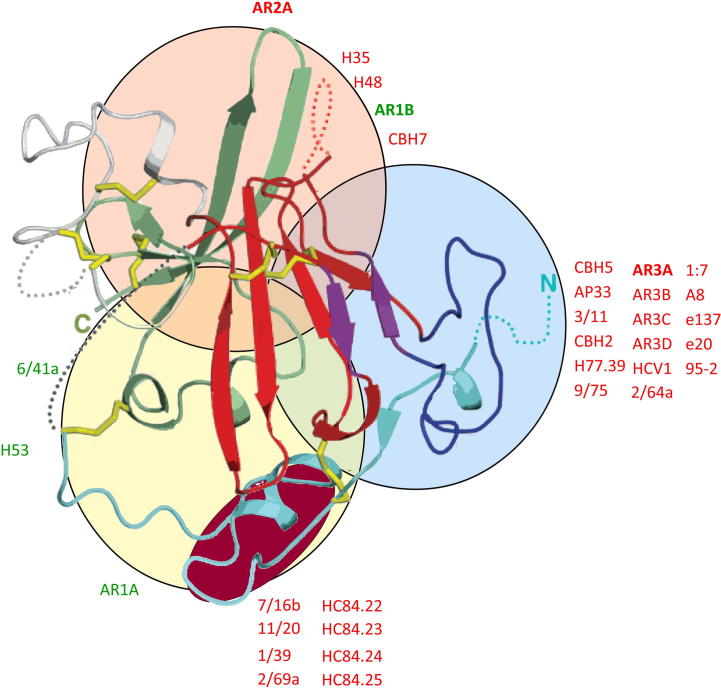
Antigenic organization of the HCV glycoprotein E2 core structure. The surface of E2 is categorized into three partially-overlapping antigenic regions, that include monoclonal antibody epitopes (labelled on the periphery of the regions) using complementary techniques of binding competition assays and, alanine scanning mutagenesis. Neutralizing antibodies are labelled in red, while non-neutralizing antibodies are labelled in green. When mapped onto the crystal structure of E2 (Kong et al., 2013), these domains highlight the CD81 binding region, possessing conserved neutralization epitopes (blue circle), a β-sheet possessing less well conserved neutralization epitopes (pink circle), and a less organized region that includes epitopes of non-neutralizing mAbs (yellow circle). An additional region containing neutralization epitopes was also revealed, that is partially formed by a helical region between aa 428–442 (burgundy oval). Disulfide bonds stabilizing the structure are highlighted in yellow, and unresolved structures within primary amino acid chain indicated by dashed lines.

**Fig. 4:**
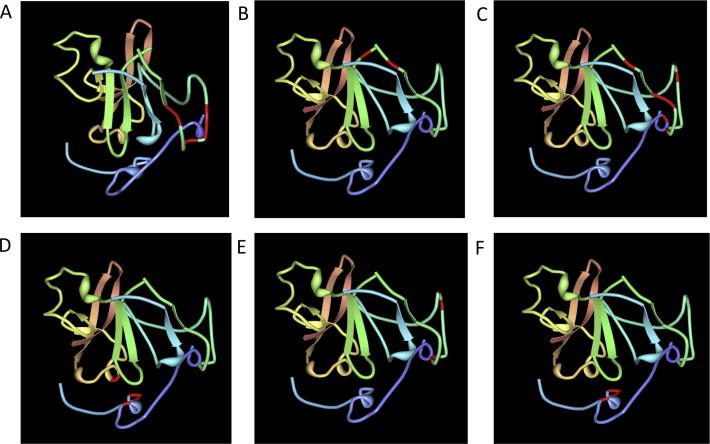
Specific residues defining receptor binding sites and antibody binding residues on the core HCV E2 glycoprotein structure. Important residues are highlighted in red on the reported structure (Kong et al., 2013) (PDB reference 4MWF). (A) Residues involved in the CD81 binding site (G523, W529, G530, D535); (B) binding residues for non-neutralizing mAb AR1B (N540, W549); (C) neutralizing mAb AR3A (S424, G523, P525, G530, D535, N540); (D) neutralizing mAb HC84.22 (N428, W437, L441, F442, Y443); (E) neutralizing mAb 1:7 (G523, W529, G530 D535); and (F) the restricted neutralizing mAb 2/69a (G440, Y443, K446). These images highlight the overlapping nature of the two discrete neutralizing epitope clusters on the core E2 structure, and the lack of neutralizing epitopes in the regions of the protein highlighted in orange, yellow and green.

The recently described E2 crystal structure (Kong et al., 2013) has provided an important framework to delineate the molecular interactions with CD81 and broadly neutralizing antibodies, confirming many of the salient points concluded from earlier receptor and antibody epitope mapping experiments. The E2 protein is mainly globular and the surface features include a neutralizing face, containing cross-reactive and strain-restricted (variable) epitopes, a highly glycosylated face and regions believed to be occluded on the intact virus particle (Fig. 3). Importantly, E2 residues previously reported play a role in CD81 binding (Owsianka et al., 2006; Tarr et al., 2006) and binding neutralizing antibodies (Johansson et al., 2007; Law et al., 2008; Owsianka et al., 2005, 2008; Perotti et al., 2008) were juxtaposed on the E2 tertiary structure, highlighting the importance of this region in antibody-based vaccine design (Fig. 4).

Recently, two groups reported the crystal structure of AP33 and HCV1 bound to their cognate peptide including aa 412–423 peptide. The key contact residues confirmed previous epitope mapping data and showed this region to adopt a beta-hairpin structure and peptide–antibody binding comprising largely hydrophobic interactions (Kong et al., 2012a,b; Potter et al., 2012), providing insights on epitope presentation that may be needed to elicit protective antibodies. Intriguingly, this region was unresolved in the recently reported E2 core structure (Kong et al., 2013), suggesting that it may be constrained by regions not present in the crystallized protein preparation – possibly HVR1, that is known to modulate the CD81 binding site and epitope exposure (Bankwitz et al., 2010). Alternatively, this region could be flexible and adopt several conformations at the virion surface, only some of which are recognized by specific neutralizing antibodies.

Structural studies of neutralizing antibodies in complex with their peptide targets is starting to reveal how some antibodies can maintain potency in the face of viral evolution. For example, analysis of human mAbs HC84-1 and HC84-27 in complex with E2 peptide aa 434–446 shows that some of the key E2 contact residues are variable. Close inspection of variant amino acids reveals that the overall property of the amino acids is conserved and this may explain the cross-reactive phenotype of these antibodies (Krey et al., 2013). However, this region contains overlapping yet distinct epitopes (Tarr et al., 2012) and amino acid variability in this region can abrogate binding of other neutralizing antibodies (Deng et al., 2013). Given these observations, designing immunogens that elicit antibodies capable of accommodating this sequence variability will be challenging. Peptide-based vaccines would be easy to produce but unconstrained peptides can adopt a myriad of conformations, only some of which would may protective antibody responses. Increasing our knowledge of the molecular and structural determinants involved in key neutralizing antibody-epitope interactions could greatly facilitate rational design of future vaccine candidates.

## 9 Future challenges and prospects

Despite the advent of increasingly effective treatments, the need for a preventive HCV vaccine has not waned. Development of antibody-based therapeutics or vaccines requires a greater understanding of the protective antibody response. A major shortfall is that much of our current understanding has been derived from studying human antibodies obtained during chronic infection or through experimental immunization of small animals. One may argue that the most protective antibodies are likely to be present during acute resolved infection (Logvinoff et al., 2004; Osburn et al., 2014; Pestka et al., 2007), yet very little is known about the nature and specificity of these antibody responses. Application of improved methods to isolate human monoclonal antibodies, including clonal sorting (Scheid et al., 2009; Smith et al., 2009; Tiller et al., 2008; Wu et al., 2010) and short-term culture (Corti et al., 2011; Walker et al., 2009), together with techniques to fractionate and analyse the polyclonal response (Li et al., 2007, 2009), will help resolve this shortfall. Functional screening of short-term B-/plasma cell cultures has the potential to identify neutralizing antibodies targeting novel epitopes; an approach that has yielded several potent anti-HIV and influenza-specific human monoclonal antibodies (Corti et al., 2011; Walker et al., 2009). This would be possible through identification of protective antibody determinants associated with acute resolved infection and by screening methods that specifically enrich/isolate antibodies targeting conserved regions of the viral glycoprotein involved in virus entry.

Immunization strategies that rely solely upon the administration of unmodified HCV subunits (e.g. E1E2 or soluble E2) are unlikely to be successful, as the variable regions are immunodominant (Puig et al., 2004). This scenario is similar to another devastating chronic viral infection, HIV-1, where current vaccine efforts focus on engineered immunogens (Ahmed et al., 2012; Pantophlet and Burton, 2003; Walker and Burton, 2010). This immune-focused approach has been facilitated by the isolation and characterisation of neutralizing human antibodies (e.g. 2F5, 2G12 and IgG-b12) that target distinct steps in the viral entry pathway (Moore et al., 1994; Pantophlet et al., 2003a,b; Saphire et al., 2001, 2002; Zwick et al., 2003). The recent elucidation of the crystal structure of HCV E2 (Kong et al., 2013) will undoubtedly pave the way for similar approaches in HCV vaccine development.

## 10 Conclusions

Studies of the HCV neutralizing response have contributed greatly to our understanding of the natural progression of hepatitis C virus infection. There is increasing evidence that the specificity and potency of the early antibody response can influence acute infection outcome and neutralizing antibodies seem to play a part in controlling HCV during chronic infection. Emerging *in vitro* and *in vivo* systems is enabling us to gain a better understanding of the antibody determinants that lead to protection. Undoubtedly, one of the major challenges for the future will be to harness this knowledge for the development of effective antibody-based vaccines and treatments.

## References

[b0005] Abe Y., Takashita E., Sugawara K., Matsuzaki Y., Muraki Y., Hongo S. (2004). Effect of the addition of oligosaccharides on the biological activities and antigenicity of influenza A/H3N2 virus hemagglutinin. J. Virol..

[b0010] Adair R., Patel A.H., Corless L., Griffin S., Rowlands D.J., McCormick C.J. (2009). Expression of hepatitis C virus (HCV) structural proteins in trans facilitates encapsidation and transmission of HCV subgenomic RNA. J. Gen. Virol..

[b0015] Ahmed F.K., Clark B.E., Burton D.R., Pantophlet R. (2012). An engineered mutant of HIV-1 gp120 formulated with adjuvant Quil A promotes elicitation of antibody responses overlapping the CD4-binding site. Vaccine.

[b0020] Aitken C.K., Lewis J., Tracy S.L., Spelman T., Bowden D.S., Bharadwaj M., Drummer H., Hellard M. (2008). High incidence of hepatitis C virus reinfection in a cohort of injecting drug users. Hepatology.

[b0025] Akazawa D., Moriyama M., Yokokawa H., Omi N., Watanabe N., Date T., Morikawa K., Aizaki H., Ishii K., Kato T., Mochizuki H., Nakamura N., Wakita T. (2013). Neutralizing antibodies induced by cell culture-derived hepatitis C virus protect against infection in mice. Gastroenterology.

[b0030] Allander T., Beyene A., Jacobson S.H., Grillner L., Persson M.A. (1997). Patients infected with the same hepatitis C virus strain display different kinetics of the isolate-specific antibody response. J. Infect. Dis..

[b0035] Amet T., Ghabril M., Chalasani N., Byrd D., Hu N., Grantham A., Liu Z., Qin X., He J.J., Yu Q. (2012). CD59 incorporation protects hepatitis C virus against complement-mediated destruction. Hepatology.

[b0040] Anjum S., Wahid A., Afzal M.S., Albecka A., Alsaleh K., Ahmad T., Baumert T.F., Wychowski C., Qadri I., Penin F., Dubuisson J. (2013). Additional glycosylation within a specific hypervariable region of subtype 3a of hepatitis C virus protects against virus neutralization. J. Infect. Dis..

[b0045] Banerjee A., Mazumdar B., Meyer K., Di Bisceglie A.M., Ray R.B., Ray R. (2011). Transcriptional repression of C4 complement by hepatitis C virus proteins. J. Virol..

[b0050] Bankwitz D., Steinmann E., Bitzegeio J., Ciesek S., Friesland M., Herrmann E., Zeisel M.B., Baumert T.F., Keck Z.Y., Foung S.K., Pecheur E.I., Pietschmann T. (2010). Hepatitis C virus hypervariable region 1 modulates receptor interactions, conceals the CD81 binding site, and protects conserved neutralizing epitopes. J. Virol..

[b0930] Barnes E., Folgori A., Capone S., Swadling L., Aston S., Kurioka A., Meyer J., Huddart R., Smith K., Townsend R., Brown A., Antrobus R., Ammendola V., Naddeo M., O’Hara G., Willberg C., Harrison A., Grazioli F., Esposito M.L., Siani L., Traboni C., Oo Y., Adams D., Hill A., Colloca S., Nicosia A., Cortese R., Klenerman P. (2012). Novel adenovirus-based vaccines induce broad and sustained T cell responses to HCV in man. Sci. Transl. Med..

[b0060] Bartosch B., Bukh J., Meunier J., Granier C., Engle R., Blackwelder W., Emerson S., Cosset F., Purcell R. (2003). In vitro assay for neutralizing antibody to hepatitis C virus: evidence for broadly conserved neutralization epitopes. Proc. Natl. Acad. Sci. USA.

[b0065] Bartosch B., Dubuisson J., Cosset F.-L. (2003). Infectious hepatitis C virus pseudo-particles containing functional E1–E2 envelope protein complexes. J. Exp. Med..

[b0070] Bartosch B., Vitelli A., Granier C., Goujon C., Dubuisson J., Pascale S., Scarselli E., Cortese R., Nicosia A., Cosset F.L. (2003). Cell entry of hepatitis C virus requires a set of co-receptors that include the CD81 tetraspanin and the SR-B1 scavenger receptor. J. Biol. Chem..

[b0075] Bassett S.E., Guerra B., Brasky K., Miskovsky E., Houghton M., Klimpel G.R., Lanford R.E. (2001). Protective immune response to hepatitis C virus in chimpanzees rechallenged following clearance of primary infection. Hepatology.

[b0080] Baumert T.F., Ito S., Wong D.T., Liang T.J. (1998). Hepatitis C virus structural proteins assemble into viruslike particles in insect cells. J. Virol..

[b0880] Baumert T.F., Wellnitz S., Aono S., Satoi J., Herion D., Tilman Gerlach J., Pape G.R., Lau J.Y., Hoofnagle J.H., Blum H.E., Liang T.J. (2000). Antibodies against hepatitis C virus-like particles and viral clearance in acute and chronic hepatitis C. Hepatology.

[b0090] Billerbeck E., de Jong Y., Dorner M., de la Fuente C., Ploss A. (2013). Animal models for hepatitis C. Curr. Top. Microbiol. Immunol..

[b0095] Bjoro K., Froland S., Yun Z., Samdal H., Haaland T. (1994). Hepatitis C infection in patients with primary hypogammaglobulinaemia after treatment with contaminated immune globulin. New Engl. J. Med..

[b0100] Blanchard E., Belouzard S., Goueslain L., Wakita T., Dubuisson J., Wychowski C., Rouille Y. (2006). Hepatitis C virus entry depends on clathrin-mediated endocytosis. J. Virol..

[b0105] Booth J.C., Kumar U., Webster D., Monjardino J., Thomas H.C. (1998). Comparison of the rate of sequence variation in the hypervariable region of E2/NS1 region of hepatitis C virus in normal and hypogammaglobulinemic patients. Hepatology.

[b0110] Brimacombe C.L., Grove J., Meredith L.W., Hu K., Syder A.J., Flores M.V., Timpe J.M., Krieger S.E., Baumert T.F., Tellinghuisen T.L., Wong-Staal F., Balfe P., McKeating J.A. (2011). Neutralising antibody-resistant hepatitis C virus cell-to-cell transmission. J. Virol..

[b0115] Broering T.J., Garrity K.A., Boatright N.K., Sloan S.E., Sandor F., Thomas W.D., Szabo G., Finberg R.W., Ambrosino D.M., Babcock G.J. (2009). Identification and characterization of broadly neutralizing human monoclonal antibodies directed against the E2 envelope glycoprotein of hepatitis C virus. J. Virol..

[b0120] Brown R.J.P., Juttla V.S., Tarr A.W., Finnis R., Irving W.L., Hemsley S., Flower D.R., Borrow P., Ball J.K. (2005). Evolutionary dynamics of hepatitis C virus envelope genes during chronic infection. J. Gen. Virol..

[b0125] Brown R.J.P., Tarr A.W., McClure C.P., Juttla V.S., Taguiri N., Irving W.L., Ball J.K. (2007). Cross-genotype characterization of genetic diversity and molecular adaptation in hepatitis C virus envelope glycoprotein genes. J. Gen. Virol..

[b0130] Burton D.R. (2002). Antibodies, viruses and vaccines. Nat. Rev. Immunol..

[b0135] Chen M., Sallberg M., Sonnerborg A., Weiland O., Mattsson L., Jin L., Birkett A., Peterson D., Milich D.R. (1999). Limited humoral immunity in hepatitis C virus infection. Gastroenterology.

[b0140] Choo Q.L., Kuo G., Ralston R., Weiner A., Chien D., Van Nest G., Han J., Berger K., Thudium K., Kuo C. (1994). Vaccination of chimpanzees against infection by the hepatitis C virus. Proc. Natl. Acad. Sci. USA.

[b0885] Chung R.T., Gordon F.D., Curry M.P., Schiano T.D., Emre S., Corey K., Markmann J.F., Hertl M., Pompiselli J., Pomfret E.A. (2013). Human monoclonal antibody MBL-HCV1 delays HCV viral rebound following liver transplantation: a randomized control study. Am. J. Transplant..

[b0150] Clayton R.F., Owsianka A., Aitken J., Graham S., Bhella D., Patel A.H. (2002). Analysis of the antigenicity and topology of E2 glycoprotein present on the recombinant hepatitis C virus-like particles. J. Virol..

[b0155] Corti D., Voss J., Gamblin S.J., Codoni G., Macagno A., Jarrossay D., Vachieri S.G., Pinna D., Minola A., Vanzetta F., Silacci C., Fernandez-Rodriguez B.M., Agatic G., Bianchi S., Giacchetto-Sasselli I., Calder L., Sallusto F., Collins P., Haire L.F., Temperton N., Langedijk J.P.M., Skehel J.J., Lanzavecchia A. (2011). A neutralizing antibody selected from plasma cells that binds to group 1 and group 2 influenza A hemagglutinins. Science.

[b0890] Davis G.L., Nelson D.R., Terrault N., Pruett T.L., Schiano T.D., Fletcher C.V., Sapan C.V., Riser L.N., Li Y., Whitley R.J., Gnann J.W., Group C.A.S. (2005). A randomized, open-label study to evaluate the safety and pharmacokinetics of human hepatitis C immune globulin (Civacir) in liver transplant recipients. Liver Transpl..

[b0165] Deng L., Zhong L., Struble E., Duan H., Ma L., Harman C., Yan H., Virata-Theimer M.L., Zhao Z., Feinstone S., Alter H., Zhang P. (2013). Structural evidence for a bifurcated mode of action in the antibody-mediated neutralization of hepatitis C virus. Proc. Natl. Acad. Sci. USA.

[b0170] Dhillon S., Witteveldt J., Gatherer D., Owsianka A.M., Zeisel M.B., Zahid M.N., Rychlowska M., Foung S.K., Baumert T.F., Angus A.G., Patel A.H. (2010). Mutations within a conserved region of the Hepatitis C virus E2 glycoprotein that influence virus-receptor interactions and sensitivity to neutralizing antibodies. J. Virol..

[b0180] Dorner M., Horwitz J.A., Robbins J.B., Barry W.T., Feng Q., Mu K., Jones C.T., Schoggins J.W., Catanese M.T., Burton D.R., Law M., Rice C.M., Ploss A. (2011). A genetically humanized mouse model for hepatitis C virus infection. Nature.

[b0175] Dorner M., Horwitz J.A., Donovan B.M., Labitt R.N., Budell W.C., Friling T., Vogt A., Catanese M.T., Satoh T., Kawai T., Akira S., Law M., Rice C.M., Ploss A. (2013). Completion of the entire hepatitis C virus life cycle in genetically humanized mice. Nature.

[b0185] Dowd K.A., Netski D.M., Wang X.H., Cox A.L., Ray S.C. (2009). Selection pressure from neutralizing antibodies drives sequence evolution during acute infection with hepatitis C virus. Gastroenterology.

[b0190] Drummer H.E., Boo I., Maerz A.L., Poumbourios P. (2006). A conserved Gly(436)-Trp-Leu-Ala-Gly-Leu-Phe-Tyr motif in hepatitis C virus glycoprotein E2 is a determinant of CD81 binding and viral entry. J. Virol..

[b0195] Duan H., Kachko A., Zhong L., Struble E., Pandey S., Yan H., Harman C., Virata-Theimer M.L., Deng L., Zhao Z., Major M., Feinstone S., Zhang P. (2012). Amino acid residue-specific neutralization and nonneutralization of hepatitis C virus by monoclonal antibodies to the E2 protein. J. Virol..

[b0200] Dubuisson J., Hsu H.H., Cheung R.C., Greenberg H.B., Russell D.G., Rice C.M. (1994). Formation and intracellular localisation of hepatitis C virus envelope glycoprotein complexes expressed by recombinant vaccinia and Sindbis viruses. J. Virol..

[b0895] Ejaz A., Steinmann E., Banki Z., Anggakusuma, Khalid S., Lengauer S., Wilhelm C., Zoller H., Schloegl A., Steinmann J., Grabski E., Kleines M., Pietschmann T., Stoiber H. (2012). Specific acquisition of functional CD59 but not CD46 or CD55 by hepatitis C virus. PLoS One.

[b0210] Elmowalid G., Qiao M., Jeong S., Borg B., Baumert T., Sapp R., Hu Z., Murthy K., Liang T. (2007). Immunization with hepatitis C virus-like particles results in control of hepatitis C virus infection in chimpanzees. Proc. Natl. Acad. Sci. USA.

[b0215] Ennishi D., Terui Y., Yokoyama M., Mishima Y., Takahashi S., Takeuchi K., Okamoto H., Tanimoto M., Hatake K. (2008). Monitoring serum hepatitis C virus (HCV) RNA in patients with HCV-infected CD20-positive B-cell lymphoma undergoing rituximab combination chemotherapy. Am. J. Hematol..

[b0220] Eren R., Landstein D., Terkieltaub D., Nussbaum O., Zauberman A., Ben-Porath J., Gopher J., Buchnick R., Kovjazin R., Rosenthal-Galili Z. (2006). Preclinical evaluation of two neutralizing human monoclonal antibodies against hepatitis C virus (HCV): a potential treatment to prevent HCV reinfection in liver transplant patients. J. Virol..

[b0230] Falkowska E., Kajumo F., Garcia E., Reinus J., Dragic T. (2007). Hepatitis C virus envelope glycoprotein E2 glycans modulate entry, CD81 binding and neutralisation. J. Virol..

[b0235] Farci P., London W.T., Wong D.C., Dawson G.J., Vallari D.S., Engle R., Purcell R.H. (1992). The natural history of infection with hepatitis C virus (HCV) in chimpanzees: comparison of serologic responses measured with first- and second-generation assays and relationship to HCV viremia. J. Infect. Dis..

[b0245] Farci P., Shimoda A., Wong D., Cabezon T., De Gioannis D., Strazzera A., Shimizu Y., Shapiro M., Alter H.J., Purcell R.H. (1996). Prevention of hepatitis C virus infection in chimpanzees by hyperimmune serum against the hypervariable region 1 of the envelope 2 protein. Proc. Natl. Acad. Sci. USA.

[b0240] Farci P., Shimoda A., Coiana A., Diaz G., Peddis G., Melpolder J.C., Strazzera A., Chien D.Y., Munoz S.J., Balestrieri A., Purcell R.H., Alter H.J. (2000). The outcome of acute hepatitis C predicted by the evolution of the viral quasispecies. Science.

[b0250] Feray C., Gigou M., Samuel D., Ducot B., Maisonneuve P., Reynes M., Bismuth A., Bismuth H. (1998). Incidence of hepatitis C in patients receiving different preparations of hepatitis B immunoglobulins after liver transplantation. Ann. Intern. Med..

[b0260] Flint M., Maidens C., Loomis-Price L.D., Shotton C., Dubuisson J., Monk P., Higginbottom A., Levy S., McKeating J. (1999). Characterisation of hepatitis C virus E2 glycoprotein interaction with a putative cellular receptor, CD81. J. Virol..

[b0265] Flint M., Thomas J.M., Maidens C.M., Shotton C., Levy S., Barclay W.S., McKeating J.A. (1999). Functional analysis of cell surface-expressed hepatitis C virus E2 glycoprotein. J. Virol..

[b0255] Flint M., Logvinoff C., Rice C.M., McKeating J.A. (2004). Characterisation of infectious retroviral pseudotype particles bearing hepatitis C virus glycoproteins. J. Virol..

[b0935] Garrone P., Fluckiger A.C., Mangeot P.E., Gauthier E., Dupeyrot-Lacas P., Mancip J., Cangialosi A., Du Chene I., LeGrand R., Mangeot I., Lavillette D., Bellier B., Cosset F.L., Tangy F., Klatzmann D., Dalba C. (2011). A prime-boost strategy using virus-like particles pseudotyped for HCV proteins triggers broadly neutralizing antibodies in macaques. Sci. Transl. Med..

[b0275] Giang E., Dorner M., Prentoe J.C., Dreux M., Evans M.J., Bukh J., Rice C.M., Ploss A., Burton D.R., Law M. (2012). Human broadly neutralizing antibodies to the envelope glycoprotein complex of hepatitis C virus. Proc. Natl. Acad. Sci. USA.

[b0280] Goffard A., Callens N., Bartosch B., Wychowski C., Cosset F.L., Montpellier C., Dubuisson J. (2005). Role of N-linked glycans in the functions of hepatitis C virus envelope glycoproteins. J. Virol..

[b0285] Gottwein J.M., Scheel T.K.H., Jensen T.B., Lademann J.B., Prentoe J.C., Knudsen M.L., Hoegh A.M., Bukh J. (2009). Development and characterisation of hepatitis C virus genotype 1–7 cell culture systems: role of CD81 and scavenger receptor class B type I and effect of antiviral drugs. Hepatology.

[b0290] Grove J., Nielsen S., Zhong J., Bassendine M.F., Drummer H.E., Balfe P., McKeating J.A. (2008). Identification of a residue in Hepatitis C virus E2 glycoprotein that determines scavenger receptor BI and CD81 receptor dependency and sensitivity to neutralising antibodies. J. Virol..

[b0295] Helle F., Goffard A., Morel V., Duverlie G., McKeating J., Keck Z.-Y., Foung S., Penin F., Dubuisson J., Voisset C. (2007). The neutralising activity of anti-hepatitis C virus antibodies is modulated by specific glycans on the E2 envelope protein. J. Virol..

[b0300] Helle F., Vieyres G., Elkrief L., Popescu C.-I., Wychowski C., Descamps V., Castelain S., Roingeard P., Duverlie G., Dubuisson J. (2010). Role of N-linked glycans in the functions of hepatitis C virus envelope proteins incorporated into infectious virions. J. Virol..

[b0305] Hessell A.J., Hangartner L., Hunter M., Havenith C.E., Beurskens F.J., Bakker J.M., Lanigan C.M., Landucci G., Forthal D.N., Parren P.W., Marx P.A., Burton D.R. (2007). Fc receptor but not complement binding is important in antibody protection against HIV. Nature.

[b0310] Hsu M., Zhang J., Flint M., Logvinoff C., Cheng-Mayer C., Rice C.M., McKeating J.A. (2003). Hepatitis C virus glycoproteins mediate pH-dependent cell entry of pseudotyped retroviral particles. Proc. Natl. Acad. Sci. USA.

[b0315] Humphreys I., Fleming V., Fabris P., Parker J., Schulenberg B., Brown A., Demetriou C., Gaudieri S., Pfafferott K., Lucas M., Collier J., Huang K.H., Pybus O.G., Klenerman P., Barnes E. (2009). Full-length characterization of hepatitis C virus subtype 3a reveals novel hypervariable regions under positive selection during acute infection. J. Virol..

[b0320] Jeong S.H., Qiao M., Nascimbeni M., Hu Z., Rehermann B., Murthy K., Liang T.J. (2004). Immunization with hepatitis C virus-like particles induces humoral and cellular immune responses in nonhuman primates. J. Virol..

[b0325] Jesudian A.B., de Jong Y.P., Jacobson I.M. (2013). Emerging therapeutic targets for hepatitis C virus infection. Clin. Gastroenterol. Hepatol..

[b0330] Johansson D.X., Voisset C., Tarr A.W., Aung M., Ball J.K., Dubuisson J., Persson M.A. (2007). Human combinatorial libraries yield rare antibodies that broadly neutralize hepatitis C virus. Proc. Natl. Acad. Sci. USA.

[b0335] Karlsson Hedestam G.B., Fouchier R.A.M., Phogat S., Burton D.R., Sodroski J., Wyatt R.T. (2008). The challenges of eliciting neutralizing antibodies to HIV-1 and to influenza virus. Nat. Rev. Microbiol..

[b0345] Kato N., Sekiya H., Ootsuyama Y., Nakazawa T., Hijikata M., Ohkoshi S., Shimotohno K. (1993). Humoral immune response to hypervariable region 1 of the putative envelope glycoprotein (gp70) of hepatitis C virus. J. Virol..

[b0340] Kato N., Ootsuyama Y., Sekiya H., Ohkoshi S., Nakazawa T., Hijikata M., Shimotohno K. (1994). Genetic drift in hypervariable region 1 of the viral genome in persistent hepatitis C virus infection. J. Virol..

[b0900] Keck Z.Y., Op De Beeck A., Hadlock K.G., Xia J., Li T.K., Dubuisson J., Foung S.K. (2004). Hepatitis C virus E2 has three immunogenic domains containing conformational epitopes with distinct properties and biological functions. J. Virol..

[b0350] Keck Z., Li T., Xia J., Bartosch B., Cosset F., Dubuisson J., Foung S. (2005). Analysis of a highly flexible conformational immunogenic domain a in hepatitis C virus E2. J. Virol..

[b0360] Keck Z.Y., Li T.K., Xia J.M., Gal-Tanamy M., Olson O., Li S.H., Patel A.H., Ball J.K., Lemon S.M., Foung S.K.H. (2008). Definition of a conserved immunodominant domain on hepatitis C virus E2 glycoprotein by neutralizing human monoclonal antibodies. J. Virol..

[b0370] Keck Z.Y., Xia J., Wang Y., Wang W., Krey T., Prentoe J., Carlsen T., Li A.Y., Patel A.H., Lemon S.M., Bukh J., Rey F.A., Foung S.K. (2012). Human monoclonal antibodies to a novel cluster of conformational epitopes on HCV E2 with resistance to neutralization escape in a genotype 2a isolate. PLoS Pathog..

[b0355] Keck Z., Wang W., Wang Y., Lau P., Carlsen T.H., Prentoe J., Xia J., Patel A.H., Bukh J., Foung S.K. (2013). Cooperativity in virus neutralization by human monoclonal antibodies to two adjacent regions located at the amino terminus of hepatitis C virus E2 glycoprotein. J. Virol..

[b0375] Kim H., Meyer K., Di Bisceglie A.M., Ray R. (2013). Hepatitis C virus suppresses C9 complement synthesis and impairs membrane attack complex function. J. Virol..

[b0385] Kong L., Giang E., Nieusma T., Robbins J.B., Deller M.C., Stanfield R.L., Wilson I.A., Law M. (2012). Structure of hepatitis C virus envelope glycoprotein E2 antigenic site 412 to 423 in complex with antibody AP33. J. Virol..

[b0390] Kong L., Giang E., Robbins J.B., Stanfield R.L., Burton D.R., Wilson I.A., Law M. (2012). Structural basis of hepatitis C virus neutralization by broadly neutralizing antibody HCV1. Proc. Natl. Acad. Sci. USA.

[b0380] Kong L., Giang E., Nieusma T., Kadam R.U., Cogburn K.E., Hua Y., Dai X., Stanfield R.L., Burton D.R., Ward A.B., Wilson I.A., Law M. (2013). Hepatitis C virus E2 envelope glycoprotein core structure. Science.

[b0395] Krey T., Meola A., Keck Z.Y., Damier-Piolle L., Foung S.K., Rey F.A. (2013). Structural basis of HCV neutralization by human monoclonal antibodies resistant to viral neutralization escape. PLoS Pathog..

[b0400] Lanford R., Guerra B., Chavez D., Bigger C., Brasky K., Wang X., Ray S., Thomas D. (2004). Cross-genotype immunity to hepatitis C virus. J. Virol..

[b0940] Lange C.M., Jacobson I.M., Rice C.M., Zeuzem S. (2014). Emerging therapies for the treatment of hepatitis C. EMBO Mol. Med..

[b0410] Lavillette D., Pecheur E.-I., Donot P., Fresquet J., Molle J., Corbau R., Dreux M., Penin F., Cosset F.L. (2007). Characterisation of fusion determinants points to the involvement of three discrete regions of both E1 and E2 glycoproteins in the membrane fusion process of hepatitis C virus. J. Virol..

[b0420] Law M., Maruyama T., Lewis J., Giang E., Tarr A.W., Stamataki Z., Gastaminza P., Chisari F.V., Jones I.M., Fox R.I., Ball J.K., McKeating J.A., Kneteman N.M., Burton D.R. (2008). Broadly neutralizing antibodies protect against hepatitis C virus quasispecies challenge. Nat. Med..

[b0415] Law J.L., Chen C., Wong J., Hockman D., Santer D.M., Frey S.E., Belshe R.B., Wakita T., Bukh J., Jones C.T., Rice C.M., Abrignani S., Tyrrell D.L., Houghton M. (2013). A hepatitis C virus (HCV) vaccine comprising envelope glycoproteins gpE1/gpE2 derived from a single isolate elicits broad cross-genotype neutralizing antibodies in humans. PLoS ONE.

[b0425] Lechmann M., Murata K., Satoi J., Vergalla J., Baumert T.F., Liang T.J. (2001). Hepatitis C virus-like particles induce virus-specific humoral and cellular immune responses in mice. Hepatology.

[b0430] Leroux-Roels G., Depla E., Hulstaert F., Tobback L., Dincq S., Desmet J., Desombere I., Maertens G. (2004). A candidate vaccine based on the hepatitis C E1 protein: tolerability and immunogenicity in healthy volunteers. Vaccine.

[b0435] Li Y., Migueles S.A., Welcher B., Svehla K., Phogat A., Louder M.K., Wu X., Shaw G.M., Connors M., Wyatt R.T., Mascola J.R. (2007). Broad HIV-1 neutralization mediated by CD4-binding site antibodies. Nat. Med..

[b0440] Li Y., Svehla K., Louder M.K., Wycuff D., Phogat S., Tang M., Migueles S.A., Wu X., Phogat A., Shaw G.M., Connors M., Hoxie J., Mascola J.R., Wyatt R. (2009). Analysis of neutralization specificities in polyclonal sera derived from human immunodeficiency virus type 1-infected individuals. J. Virol..

[b0445] Lindenbach B., Evans M., Syder A., Wolk B., Tellinghuisen T., Liu C., Maruyama T., Hynes R., Burton D., McKeating J., Rice C. (2005). Complete replication of hepatitis C virus in cell culture. Science.

[b0450] Liu L., Fisher B.E., Dowd K.A., Astemborski J., Cox A.L., Ray S.C. (2010). Acceleration of hepatitis C virus envelope evolution in humans is consistent with progressive humoral immune selection during the transition from acute to chronic infection. J. Virol..

[b0455] Logvinoff C., Major M., Oldach D., Heyward S., Talal A., Balfe P., Feinstone S., Alter H., Rice C., McKeating J. (2004). Neutralizing antibody response during acute and chronic hepatitis C virus infection. Proc. Natl. Acad. Sci. USA.

[b0460] Machida K., Kondo Y., Huang J.Y., Chen Y.C., Cheng K.T., Keck Z., Foung S., Dubuisson J., Sung V.M., Lai M.M. (2008). Hepatitis C virus (HCV)-induced immunoglobulin hypermutation reduces the affinity and neutralizing activities of antibodies against HCV envelope protein. J. Virol..

[b0465] Mailly L., Robinet E., Meuleman P., Baumert T.F., Zeisel M.B. (2013). Hepatitis C virus infection and related liver disease: the quest for the best animal model. Front Microbiol..

[b0475] Mazumdar B., Kim H., Meyer K., Bose S.K., Di Bisceglie A.M., Ray R.B., Ray R. (2012). Hepatitis C virus proteins inhibit C3 complement production. J. Virol..

[b0470] Mazumdar B., Kim H., Meyer K., Bose S.K., Di Bisceglie A.M., Ray R.B., Diamond M.S., Atkinson J.P., Ray R. (2013). Hepatitis C virus infection upregulates CD55 expression on the hepatocyte surface and promotes association with virus particles. J. Virol..

[b0480] Meertens L., Bertaux C., Dragic T. (2006). Hepatitis C virus entry requires a critical postinternalization step and delivery to early endosomes via clathrin-coated vesicles. J. Virol..

[b0485] Mehta S.H., Cox A., Hoover D.R., Wang X.H., Mao Q., Ray S., Strathdee S.A., Vlahov D., Thomas D.L. (2002). Protection against persistence of hepatitis C. Lancet.

[b0490] Mercer D.F., Schiller D.E., Elliot J.F., Douglas D.N., Hao C., Rinfret A., Addison W.R., Fischer K.P., Churchill T.A., Lakey J.R.T., Tyrrell D.L.J., Kneteman N.M. (2001). Hepatitis C virus replication in mice chimeric human livers. Nat. Med..

[b0495] Meredith L.W., Wilson G.K., Fletcher N.F., McKeating J.A. (2012). Hepatitis C virus entry: beyond receptors. Rev. Med. Virol..

[b0500] Meuleman P., Bukh J., Verhoye L., Farhoudi A., Vanwolleghem T., Wang R.Y., Desombere I., Alter H., Purcell R.H., Leroux-Roels G. (2011). In vivo evaluation of the cross-genotype neutralizing activity of polyclonal antibodies against hepatitis C virus. Hepatology.

[b0505] Meunier J.C., Russell R.S., Goossens V., Priem S., Walter H., Depla E., Union A., Faulk K.N., Bukh J., Emerson S.U., Purcell R.H. (2008). Isolation and characterization of broadly neutralizing human monoclonal antibodies to the E1 glycoprotein of hepatitis C virus. J. Virol..

[b0515] Meyer K., Basu A., Przysiecki C.T., Lagging L.M., Di Bisceglie A.M., Conley A.J., Ray R. (2002). Complement-mediated enhancement of antibody function for neutralization of pseudotype virus containing hepatitis C virus E2 chimeric glycoprotein. J. Virol..

[b0510] Meyer K., Ait-Goughoulte M., Keck Z.Y., Foung S., Ray R. (2008). Antibody-dependent enhancement of hepatitis C virus infection. J. Virol..

[b0520] Mondelli M.U., Cerino A., Segagni L., Meola A., Cividini A., Silini E., Nicosia A. (2001). Hypervariable region 1 of hepatitis C virus: immunological decoy or biologically relevant domain?. Antiviral Res..

[b0525] Moore J., Sattentau Q., Wyatt R., Sodroski J. (1994). Probing the structure of the human immunodeficiency virus surface glycoprotein gp120 with a panel of monoclonal antibodies. J. Virol..

[b0530] Morin T.J., Broering T.J., Leav B.A., Blair B.M., Rowley K.J., Boucher E.N., Wang Y., Cheslock P.S., Knauber M., Olsen D.B., Ludmerer S.W., Szabo G., Finberg R.W., Purcell R.H., Lanford R.E., Ambrosino D.M., Molrine D.C., Babcock G.J. (2012). Human monoclonal antibody HCV1 effectively prevents and treats HCV infection in chimpanzees. PLoS Pathog..

[b0535] Mothes W., Sherer N.M., Jin J., Zhong P. (2010). Virus cell-to-cell transmission. J. Virol..

[b0540] Murata K., Lechmann M., Qiao M., Gunji T., Alter H.J., Liang T.J. (2003). Immunization with hepatitis C virus-like particles protects mice from recombinant hepatitis C virus-vaccinia infection. Proc. Natl. Acad. Sci. USA.

[b0545] Nattermann J., Schneiders A.M., Leifeld L., Langhans B., Schulz M., Inchauspe G., Matz B., Brackmann H.H., Houghton M., Sauerbruch T., Spengler U. (2005). Serum antibodies against the hepatitis C virus E2 protein mediate antibody-dependent cellular cytotoxicity (ADCC). J. Hepatol..

[b0550] Neumann-Haefelin C., Thimme R. (2013). Adaptive immune responses in hepatitis C virus infection. Curr. Top. Microbiol. Immunol..

[b0905] Nevens F., Roskams T., Van Vlierberghe H., Horsmans Y., Sprengers D., Elewaut A., Desmet V., Leroux-Roels G., Quinaux E., Depla E., Dincq S., Vander Stichele C., Maertens G., Hulstaert F. (2003). A pilot study of therapeutic vaccination with envelope protein E1 in 35 patients with chronic hepatitis C. Hepatology.

[b0560] Ni Y.H., Chang M.H., Chen P.J., Hsu H.Y., Lu T.W., Lin K.H., Lin D.T. (1999). Decreased diversity of hepatitis C virus quasispecies during bone marrow transplantation. J. Med. Virol..

[b0945] Osburn W.O., Snider A.E., Wells B.L., Latanich R., Bailey J.R., Thomas D.L., Cox A.L., Ray S.C. (2014). Clearance of hepatitis C infection is associated with early appearance of broad neutralizing antibody responses. Hepatology.

[b0570] Owsianka A., Clayton R.F., Loomis-Price L.D., McKeating J., Patel A.H. (2001). Functional analysis of hepatitis C virus E2 glycoproteins and virus-like particles reveals structural dissimilarities between different forms of E2. J. Gen. Virol..

[b0575] Owsianka A., Tarr A.W., Juttla V.S., Lavillette D., Bartosch B., Cosset F.L., Ball J.K., Patel A.H. (2005). Monoclonal antibody AP33 defines a broadly neutralizing epitope on the hepatitis C virus E2 envelope glycoprotein. J. Virol..

[b0585] Owsianka A.M., Timms J.M., Tarr A.W., Brown R.J.P., Hickling T.P., Szwejk A., Bienkowska-Szewczyk K., Thomson B.J., Patel A.H., Ball J.K. (2006). Identification of conserved residues in the E2 envelope glycoprotein of the hepatitis C virus that are critical for CD81 binding. J. Virol..

[b0580] Owsianka A., Tarr A.W., Keck Z.-Y., Li T.-K., Witteveldt J., Adair R., Foung S.K.H., Ball J.K., Patel A.H. (2008). Broadly neutralising human monoclonal antibodies to the hepatitis C virus E2 glycoprotein. J. Gen. Virol..

[b0590] Pantophlet R., Burton D.R. (2003). Immunofocusing: antigen engineering to promote the induction of HIV-neutralizing antibodies. Trends Mol. Med..

[b0600] Pantophlet R., Wilson I.A., Burton D.R. (2003). Hyperglycosylated mutants of human immunodeficiency virus (HIV) type 1 monomeric gp120 as novel antigens for HIV vaccine design. J. Virol..

[b0910] Pantophlet R., Ollmann Saphire E., Poignard P., Parren P., Wilson I.A., Burton D.R. (2003). Fine mapping of the interaction of neutralizing and nonneutralizing monoclonal antibodies with the CD4 binding site of human immunodeficiency virus type 1 gp120. J. Virol..

[b0605] Pantua H., Diao J., Ultsch M., Hazen M., Mathieu M., McCutcheon K., Takeda K., Date S., Cheung T.K., Phung Q., Hass P., Arnott D., Hongo J.A., Matthews D.J., Brown A., Patel A.H., Kelley R.F., Eigenbrot C., Kapadia S.B. (2013). Glycan shifting on hepatitis C virus (HCV) E2 glycoprotein is a mechanism for escape from broadly neutralizing antibodies. J. Mol. Biol..

[b0950] Pedersen J., Carlsen T.H., Prentoe J., Ramirez S., Jensen T.B., Forns X., Alter H., Foung S.K., Law M., Gottwein J., Weis B., Bukh J. (2013). Neutralization resistance of hepatitis C virus can be overcome by recombinant human monoclonal antibodies. Hepatology.

[b0615] Perotti M., Mancini N., Diotti R.A., Tarr A.W., Ball J.K., Owsianka A., Adair R., Patel A.H., Clementi M., Burioni R. (2008). Identification of a broadly cross-reacting and neutralizing human monoclonal antibody directed against the hepatitis C virus E2 protein. J. Virol..

[b0620] Pestka J.M., Zeisel M.B., Blaser E., Schurmann P., Bartosch B., Cosset F.-L., Patel A.H., Meisel H., Baumert J., Viazov S., Rispeter K., Blum H.E., Roggendorf M., Baumert T.F. (2007). Rapid induction of virus-neutralising antibodies and viral clearance in a single-source outbreak of hepatitis C. Proc. Natl. Acad. Sci. USA.

[b0625] Pietschmann T., Kaul A., Koutsoudakis G., Shavinskaya A., Kallis S., Steinmann E., Abid K., Negro F., Dreux M., Cosset F., Bartenschlager R. (2006). Construction and characterization of infectious intragenotypic and intergenotypic hepatitis C virus chimeras. Proc. Natl. Acad. Sci. USA.

[b0630] Potter J.A., Owsianka A.M., Jeffery N., Matthews D.J., Keck Z.Y., Lau P., Foung S.K., Taylor G.L., Patel A.H. (2012). Toward a hepatitis C virus vaccine: the structural basis of hepatitis C virus neutralization by AP33, a broadly neutralizing antibody. J. Virol..

[b0635] Prentoe J., Jensen T.B., Meuleman P., Serre S.B.N., Scheel T.K.H., Leroux-Roels G., Gottwein J.M., Bukh J. (2011). Hypervariable region 1 differentially impacts viability of hepatitis C virus strains of genotypes 1 to 6 and impairs virus neutralisation. J. Virol..

[b0640] Puig M., Major M.E., Mihalik K., Feinstone S.M. (2004). Immunization of chimpanzees with an envelope protein-based vaccine enhances specific humoral and cellular immune responses that delay hepatitis C virus infection. Vaccine.

[b0645] Qiao M., Murata K., Davis A.R., Jeong S.H., Liang T.J. (2003). Hepatitis C virus-like particles combined with novel adjuvant systems enhance virus-specific immune responses. Hepatology.

[b0650] Raghuraman S., Park H., Osburn W.O., Winkelstein E., Edlin B.R., Rehermann B. (2012). Spontaneous clearance of chronic hepatitis C virus infection is associated with appearance of neutralizing antibodies and reversal of T-cell exhaustion. J. Infect. Dis..

[b0655] Ray S.C., Wang Y.-M., Laeyendecker O., Ticehurst J.R., Villano S.A., Thomas D.L. (1999). Acute hepatitis C virus structural gene sequences as predictors of persistent viremia: hypervariable region 1 as a decoy. J. Virol..

[b0660] Rehermann B. (2013). Pathogenesis of chronic viral hepatitis: differential roles of T cells and NK cells. Nat. Med..

[b0665] Rosa D., Campagnoli S., Moretto C., Guenzi E., Cousens L., Chin M., Dong C., Weiner A.J., Lau J.Y.N., Choo Q.L., Chien D., Pileri P., Houghton M., Abrignani S. (1996). A quantitative test to estimate neutralising antibodies to the hepatitis C virus: Cytofluorimetric assessment of envelope glycoprotein 2 binding to target cells. Proc. Natl. Acad. Sci. USA.

[b0670] Rothwangl K.B., Manicassamy B., Uprichard S.L., Rong L. (2008). Dissecting the role of putative CD81 binding regions of E2 in mediating HCV entry: putative CD81 binding region 1 is not involved in CD81 binding. Virol. J..

[b0675] Sabo M.C., Luca V.C., Prentoe J., Hopcraft S.E., Blight K.J., Yi M., Lemon S.M., Ball J.K., Bukh J., Evans M.J., Fremont D.H., Diamond M.S. (2011). Neutralizing monoclonal antibodies against hepatitis C virus E2 protein bind discontinuous epitopes and inhibit infection at a postattachment step. J. Virol..

[b0680] Saphire E.O., Parren P., Pantophlet R., Zwick M.B., Morris G.M., Rudd P.M., Dwek R.A., Stanfield R.L., Burton D.R., Wilson I.A. (2001). Crystal structure of a neutralizing human IgG against HIV-1: A template for vaccine design. Science.

[b0685] Saphire E.O., Stanfield R.L., Crispin M.D.M., Parren P., Rudd P.M., Dwek R.A., Burton D.R., Wilson I.A. (2002). Contrasting IgG structures reveal extreme asymmetry and flexibility. J. Mol. Biol..

[b0690] Scarselli E., Ansuini H., Cerino R., Roccasecca R.M., Acali S., Filocamo G., Traboni C., Nicosia A., Cortese R., Vitelli A. (2002). The human scavenger receptor class B type I is a novel candidate receptor for the hepatitis C virus. Embo J..

[b0695] Scheid J.F., Mouquet H., Feldhahn N., Seaman M.S., Velinzon K., Pietzsch J., Ott R.G., Anthony R.M., Zebroski H., Hurley A., Phogat A., Chakrabarti B., Li Y., Connors M., Pereyra F., Walker B.D., Wardemann H., Ho D., Wyatt R.T., Mascola J.R., Ravetch J.V., Nussenzweig M.C. (2009). Broad diversity of neutralizing antibodies isolated from memory B cells in HIV-infected individuals. Nature.

[b0700] Schiano T.D., Charlton M., Younossi Z., Galun E., Pruett T., Tur-Kaspa R., Eren R., Dagan S., Graham N., Williams P.V., Andrews J. (2006). Monoclonal antibody HCV-AbXTL68 in patients undergoing liver transplantation for HCV: Results of a phase 2 randomized study. Liver Transpl..

[b0705] Shimizu Y.K., Hijikata M., Iwamoto A., Alter H.J., Purcell R.H., Yoshikura H. (1994). Neutralizing antibodies against hepatitis C virus and the emergence of neutralization escape mutant viruses. J. Virol..

[b0710] Shui J., Hwang W.C., Perez S., Wei G., Aird D., Chen L.-M., Santelli E., Stec B., Cadwell G., Ali M., Wan H., Murakami A., Yammanuru A., Han T., Cox N.J., Bankston L.A., Donis R.O., Liddington R.C., Marasco W.A. (2009). Structural and functional bases for broad-spectrum neutralisation of avian and human influenza A viruses. Nat. Struct. Mol. Biol..

[b0715] Simmonds P. (2004). Genetic diversity and evolution of hepatitis C virus – 15 years on. J. Gen. Virol..

[b0720] Smith K., Garman L., Wrammert J., Zheng N.-Y., Capra J.D., Ahmed R., Wilson P.C. (2009). Rapid generation of fully human monoclonal antibodies specific to a vaccinating antigen. Nat. Protocols.

[b0730] Stamataki Z., Coates S., Evans M.J., Wininger M., Crawford K., Dong C., Fong Y.-L., Chien D., Abrignani S., Balfe P., Rice C.M., McKeating J.A., Houghton M. (2007). Hepatitis C virus envelope glycoprotein immunization of rodents elicits cross-reactive neutralizing antibodies. Vaccine.

[b0725] Stamataki Z., Coates S., Abrignani S., Houghton M., McKeating J.A. (2011). Immunization of human volunteers with hepatitis C virus envelope glycoproteins elicits antibodies that cross-neutralize heterologous virus strains. J. Infect. Dis..

[b0735] Sung V.M., Shimodaira S., Doughty A.L., Picchio G.R., Can H., Yen T.S., Lindsay K.L., Levine A.M., Lai M.M. (2003). Establishment of B-cell lymphoma cell lines persistently infected with hepatitis C virus in vivo and in vitro: the apoptotic effects of virus infection. J. Virol..

[b0750] Tarr A.W., Owsianka A.M., Timms J.M., McClure C.P., Brown R.J.P., Hickling T.P., Pietschmann T., Bartenschlager R., Patel A.H., Ball J.K. (2006). Characterization of the hepatitis C virus E2 epitope defined by the broadly neutralizing monoclonal antibody AP33. Hepatology.

[b0745] Tarr A.W., Owsianka A.M., Jayaraj D., Brown R.J.P., Hickling T.P., Irving W.L., Patel A.H., Ball J.K. (2007). Determination of the human antibody response to the epitope defined by the hepatitis C virus neutralising monoclonal antibody AP33. J. Gen. Virol..

[b0755] Tarr A.W., Urbanowicz R.A., Hamed M.R., Albecka A., McClure C.P., Brown R.J., Irving W.L., Dubuisson J., Ball J.K. (2011). Hepatitis C patient-derived glycoproteins exhibit marked differences in susceptibility to serum neutralizing antibodies: genetic subtype defines antigenic but not neutralization serotype. J. Virol..

[b0760] Tarr A.W., Urbanowicz R.A., Jayaraj D., Brown R.J., McKeating J.A., Irving W.L., Ball J.K. (2012). Naturally occurring antibodies that recognize linear epitopes in the amino terminus of the hepatitis C virus E2 protein confer noninterfering, additive neutralization. J. Virol..

[b0740] Tarr A.W., Lafaye P., Meredith L., Damier-Piolle L., Urbanowicz R.A., Meola A., Jestin J.L., Brown R.J., McKeating J.A., Rey F.A., Ball J.K., Krey T. (2013). An alpaca nanobody inhibits hepatitis C virus entry and cell-to-cell transmission. Hepatology.

[b0765] Tiller T., Meffre E., Yurasov S., Tsuiji M., Nussenzweig M.C., Wardemann H. (2008). Efficient generation of monoclonal antibodies from single human B cells by single cell RT-PCR and expression vector cloning. J. Immunol. Meth..

[b0915] Timpe J.M., Stamataki Z., Jennings A., Hu K., Farquhar M.J., Harris H.J., Schwarz A., Desombere I., Leroux Roels G., Balfe P., McKeating J.A. (2007). Hepatitis C virus cell–cell transmission in hepatoma cells in the presence of neutralizing antibodies. Hepatology.

[b0775] van Doorn L.J., Kleter G.E., Stuyver L., Maertens G., Brouwer J.T., Schalm S.W., Heijtink R.A., Quint W.G. (1995). Sequence analysis of hepatitis C virus genotypes 1–5 reveals multiple novel subtypes in the Benelux countries. J. Gen. Virol..

[b0780] Vanwolleghem T., Bukh J., Meuleman P., Desombere I., Meunier J.C., Alter H., Purcell R.H., Leroux-Roels G. (2008). Polyclonal immunoglobulins from a chronic hepatitis C virus patient protect human liver-chimeric mice from infection with a homologous hepatitis C virus strain. Hepatology.

[b0785] Verstrepen B.E., Depla E., Rollier C.S., Mares G., Drexhage J.A.R., Priem S., Verschoor E.J., Koopman G., Granier C., Dreux M., Cosset F.L., Maertens G., Heeney J.L. (2011). Clearance of genotype 1b hepatitis C virus in chimpanzees in the presence of vaccine-induced E1-neutralising antibodies. J. Infect. Dis..

[b0790] von Hahn T., Yoon J.C., Alter H., Rice C.M., Rehermann B., Balfe P., McKeating J.A. (2007). Hepatitis C virus continuously escapes from neutralizing antibody and T-cell responses during chronic infection in vivo. Gastroenterology.

[b0795] Wakita T., Pietschmann T., Kato T., Date T., Miyamoto M., Zhao Z., Murthy K., Habermann A., Krausslich H.G., Mizokami M., Bartenschlager R., Liang T.J. (2005). Production of infectious hepatitis C virus in tissue culture from a cloned viral genome. Nat. Med..

[b0800] Walker L.M., Burton D.R. (2010). Rational antibody-based HIV-1 vaccine design: current approaches and future directions. Cur. Opin. Immunol..

[b0805] Walker L.M., Phogat S.K., Chan-Hui P.-Y., Wagner D., Phung P., Goss J.L., Wrin T., Simek M.D., Fling S., Mitcham J.L., Lehrman J.K., Priddy F.H., Olsen O.A., Frey S.M., Hammond P.W., Investigators P.G.P., Kaminsky S., Zamb T., Moyle M., Koff W.C., Poignard P., Burton D.R. (2009). Broad and potent neutralizing antibodies from an African donor reveal a new HIV-1 vaccine target. Science.

[b0810] Wei X., Decker J.M., Wang S., Huxiong H., Kappes J.C., Wu X., Salazar-Gonzalez J.F., Salazar M.G., Kilby J.M., Saag M.S., Komarova N.L., Nowak M.A., Hahn B.H., Kwong P.D., Shaw G.M. (2003). Antibody neutralisation and escape by HIV-1. Nature.

[b0820] Weiner A.J., Geysen H.M., Christopherson C., Hall J.E., Mason T.J., Saracco G., Bonino F., Crawford K., Marion C.D., Crawford K.A., Brunetto M., Barr P.J., Miyamura T., McHutchinson J., Houghton M. (1992). Evidence for immune selection of hepatitis C virus (HCV) putative envelope glycoprotein variants: potential role in chronic HCV infections. Proc. Natl. Acad. Sci. USA.

[b0815] Weiner A., Paliard X., Selby M., Medina-Selby A., Coit D., Nguyen S., Kansopon J., Arian C., Ng P., Tucker J., Lee C., Polakos N., Han J., Wong S., Lu H., Rosenberg S., Brasky K., Chien D., Kuo G., Houghton M. (2001). Intrahepatic genetic inoculation of hepatitis C virus RNA confers cross-protective immunity. J. Virol..

[b0825] Witteveldt J., Evans M.J., Bitzegeio J., Koutsoudakis G., Owsianka A.M., Angus A.G.N., Keck Z.-Y., Foung S.K.H., Pietschmann T., Rice C.M., Patel A.H. (2009). CD81 is dispensable for hepatitis C virus cell-to-cell transmission in hepatoma cells. J. Gen. Virol..

[b0830] Wu X., Yang Z.-Y., Li Y., Hogerkorp C.-M., Schief W.R., Seaman M.S., Zhou T., Schmidt S.D., Wu L., Xu L., Longo N.S., McKee K., O’Dell S., Louder M.K., Wycuff D.L., Feng Y., Nason M., Doria-Rose N., Connors M., Kwong P.D., Roederer M., Wyatt R.T., Nabel G.J., Mascola J.R. (2010). Rational design of envelope identifies broadly neutralizing human monoclonal antibodies to HIV-1. Science.

[b0835] Youn J., Park S., Lavillette D., Cosset F., Yang S., Lee C., Jin H., Kim C., Shata M., Lee D., Pfahler W., Prince A., Sung Y. (2005). Sustained E2 antibody response correlates with reduced peak viremia after hepatitis C virus infection in the chimpanzee. Hepatology.

[b0840] Yu M.-Y.W., Bartosch B., Zhang P., Guo Z.-P., Renzi P.M., Shen L.-M., Granier C., Feinstone S.M., Cosset F.L., Purcell R.H. (2004). Neutralizing antibodies to hepatitis C virus (HCV) in immune globulins derived from anti-HCV-positive plasma. Proc. Natl. Acad. Sci. USA.

[b0845] Zeisel M.B., Felmlee D.J., Baumert T.F. (2013). Hepatitis C virus entry. Curr. Top. Microbiol. Immunol..

[b0920] Zhang P., Wu C.G., Mihalik K., Virata-Theimer M.L., Yu M.-Y.W., Alter H.J., Feinstone S.M. (2007). Hepatitis C virus epitope specific neutralizing antibodies in Igs prepared from human plasma. Proc. Natl. Acad. Sci. USA.

[b0925] Zhang P., Zhong L., Budo Struble E., Watanabe H., Kachko A., Mihalik K., Virata-Theimer M.L., Alter H.J., Feinstone S., Major M. (2009). Depletion of interfering antibodies in chronic hepatitis C patients and vaccinated chimpanzees reveals broad cross-genotype neutralizing activity. Proc. Natl. Acad. Sci. USA.

[b0860] Zhong J., Gastaminza P., Cheng G., Kapadia S., Kato T., Burton D.R., Wieland S.F., Uprichard S.L., Wakita T., Chisari F.V. (2005). Robust hepatitis C virus infection in vitro. Proc. Natl. Acad. Sci. USA.

[b0865] Zibert A., Meisel H., Kraas W., Schulz A., Jung G., Roggendorf M. (1997). Early antibody response against hypervariable region 1 is associated with acute self-limiting infections of hepatitis C virus. Hepatology.

[b0870] Zucchelli S., Roccasecca R., Meola A., Ercole B.B., Tafi R., Dubuisson J., Galfre G., Cortese R., Nicosia A. (2001). Mimotopes of the hepatitis C virus hypervariable region 1, but not the natural sequences, induce cross-reactive antibody response by genetic immunization. Hepatology.

[b0875] Zwick M.B., Parren P., Saphire E.O., Church S., Wang M., Scott J.K., Dawson P.E., Wilson I.A., Burton D.R. (2003). Molecular features of the broadly neutralizing immunoglobulin G1 b12 required for recognition of human immunodeficiency virus type 1 gp120. J. Virol..

